# Synergistic Effects of Carrot ( *Daucus carota* L.) Root and Papaya ( *Carica papaya* L.) Extracts in Combating Inflammation and Enhancing Immune Functions—A New Perspective

**DOI:** 10.1002/fsn3.70313

**Published:** 2025-05-23

**Authors:** Muhammad Tayyab Arshad, Sammra Maqsood, Ali Ikram, Muhammed Adem Abdullahi

**Affiliations:** ^1^ University Institute of Food Science and Technology The University of Lahore Lahore Pakistan; ^2^ National Institute of Food Science and Technology University of Agriculture Faisalabad Pakistan; ^3^ Department of Food Science and Postharvest Technology, Jimma University College of Agriculture and Veterinary Medicine Jimma University Jimma Ethiopia

**Keywords:** antioxidant, carotenoids, flavonoids, fruit

## Abstract

Papaya and carrot root extracts are increasingly recognized for their ability to modulate inflammation and modify immunological functions due to their richness in bioactive compounds. Beta‐carotene, polyphenols, and flavonoids, which have antioxidant effects, have been reported to induce the activation of immune cells while reducing oxidative stress and are also abundant in carrot root. These substances help suppress proinflammatory cytokines like TNF‐α and IL‐6 and regulate inflammatory pathways like NF‐κB. Papaya extracts have potent anti‐inflammatory and immunomodulatory properties since they contain vitamins A, C, and E and enzymes like papain. Bioactive compounds in the papaya, like flavonoids and lycopene, balance cytokine output, improve innate immune responses, and reduce signs of inflammation in acute and chronic conditions. This paper examines the processes involved with papaya and carrot root extracts and their actions on inflammation and immunological function. It also discusses any possible synergistic effects that may result from combining them. Further, we detail how they are used in nutraceuticals and functional meals to help treat inflammation and immunological function problems. Preclinical and clinical research studies and research gaps supplement these. The review outlines difficulties in standardizing the extracts for medical use and calls for further clinical studies.

## Graphical abstract

Our paper underscores the contributions of key compounds, such as beta‐carotene, polyphenols, flavonoids, and vitamins, in suppressing pro‐inflammatory cytokines (e.g., TNF‐α, IL‐6) and regulating critical inflammatory pathways, such as NF‐κB. The synergistic properties of papaya and carrot root extracts, alongside their potential applications in nutraceuticals and functional foods, are detailed with a focus on preclinical and clinical findings. Furthermore, we address the challenges of standardizing these extracts for medical use and highlight the need for advanced studies on bioavailability, mechanistic pathways, and therapeutic innovation.

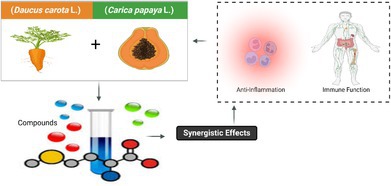

## 1 Introduction

The immune system guards the body against infections, diseases, and other pathogens; it acts as a dynamic and complex system of cells, tissues, and organs (Zmora et al. 2017). It must recognize and kill infections while distinguishing them from the body's healthy cells to maintain homeostasis (Patil et al. 2021). Besides defense against infections, good immune function is also needed to regulate inflammatory responses and maintain metabolic integrity (Calder et al. 2020). Deficiencies in essential nutrients such as vitamins and minerals can impair immunity. Hence, nutrition plays a significant role in immunological functioning (Childs et al. 2019; Weyh et al. 2022). Overall health depends on the balance of immunology, and interventions that go as simple as changes in diet can make a massive difference in overall immune activity (Walsh 2018; Di Sotto et al. 2020). Calabrese and Fasenmyer (2017) report that inflammation plays a critical role in the immune response and acts as a protective mechanism against infection or injury by facilitating healing and repair. However, chronic inflammation can lead to tissue damage and is therefore implicated in the causation of many diseases, among them cancer, metabolic syndromes, and autoimmune disorders (Pathak et al. 2022; Jiang et al. 2021).

Plant‐based nutraceuticals, such as those produced from papaya and carrots, present great potential in anti‐inflammatory and immunomodulatory therapy because they enhance the immune system and affect unique inflammation pathways (Baraya et al. 2017; Olayem et al. 2024). Such natural compounds can provide adjunctive means to conventional therapeutic approaches, mitigating the adverse impact of inflammation and increasing immunological resistance (Husen and Iqbal 2021; Alhazmi et al. 2021). Given the broad scope that plant extracts can have in influencing the immune mechanism and modulating inflammatory reactions, interest in them has grown worldwide. The benefits of these plant products in immunomodulation and anti‐inflammation, without any of the side effects sometimes encountered with manmade medicines, are more clearly evident as further information on them comes to light (Zmora et al. 2017; Patil et al. 2021). In order to be strongly immune, one has to have proper nutritional status, which is best obtained through plant diets (Calder et al. 2020). Benefiting immunological response and overall health, these bioactive phytochemicals in the plant—involving carotenoids, flavonoids, vitamins, and minerals—possess anti‐inflammatory and antioxidant activities (Childs et al. 2019; Weyh et al. 2022). For example, it was demonstrated that phytochemicals from papayas and carrots increase immune function while scavenging free radicals that worsen inflammation (Yusuf et al. 2021; Sharma et al. 2012). Although chronic inflammation is an additive factor to many diseases, such as diabetes and cardiovascular and neurological disorders, inflammation is an innate body response (Arshad et al. 2025; Ikram et al. 2024; Agarwal and Shanmugam 2022). Increasingly, nutraceuticals derived from plants have shown potential in modulating proinflammatory cytokines and pathways and have become therapeutic alternatives in place of pharmaceutical drugs (Pathak et al. 2022). For example, carrot roots serve as a functional food in treating inflammatory diseases; they contain antioxidants and phenolic compounds that reduce inflammatory indicators (Blando et al. 2021; Mizgier et al. 2016).

Abundant sources of β‐carotene, polyphenols, and anthocyanins, carrot roots can enhance immune cell function through the modulation of phagocytosis and cytokines besides antioxidant activity (Que et al. 2019; Bhandari et al. 2022). On the other hand, papaya ( *Carica papaya* ) is a rich source of flavonoids, papain enzyme, vitamins A and C, with good immune‐boosting and anti‐inflammatory properties (Khor et al. 2021; Agada et al. 2021). Papaya extracts have been identified to suppress the NF‐κB signaling pathway responsible for most chronic inflammation (Uribe et al. 2015; Vega‐Gálvez et al. 2019). Exactly how these plant extracts function at the mechanisms is something that is at present under active investigation. Jiang et al. (2021) and Mandrich et al. (2023) say that the compounds in carrots and papayas have an inhibitory action on proinflammatory mediators like interleukin‐6 (IL‐6) and tumor necrosis factor‐alpha (TNF‐α). This, in turn, affects macrophage activity and T‐cell differentiation. As distinguished by Calabrese and Fasenmyer (2017) and Alhazmi et al. (2021), these structures specify that these might be a great advantage in handling autoimmune diseases and infectious illnesses. Plant‐derived immunomodulators are progressively well documented for their frequent welfare and capability to accompany the body's natural metabolic roles. Other than reducing side effects, their solicitation in nutritional and beneficial parts has the capability to moderate immune‐associated syndromes (Jantan et al. 2015; Husen and Iqbal 2021). Zmora et al. (2017) and Calder et al. (2020) described that further investigations on the bioavailability, dose, and synergistic interactions of plant‐derived compounds are needed for their optimal use in immunological health and inflammation control.

Excerpts of papaya ( *Carica papaya* ) and carrot root ( *Daucus carota* ) play diverse functions in moderating the immune system and inflammatory decrease, as emphasized in this paper. Bioactive composites of binary plants have diverse molecular and chemical mechanisms through which these interrelate with inflammation‐associated paths and immunological procedures.

## 2 Nutritional Composition of Carrot

**FIGURE 1:**
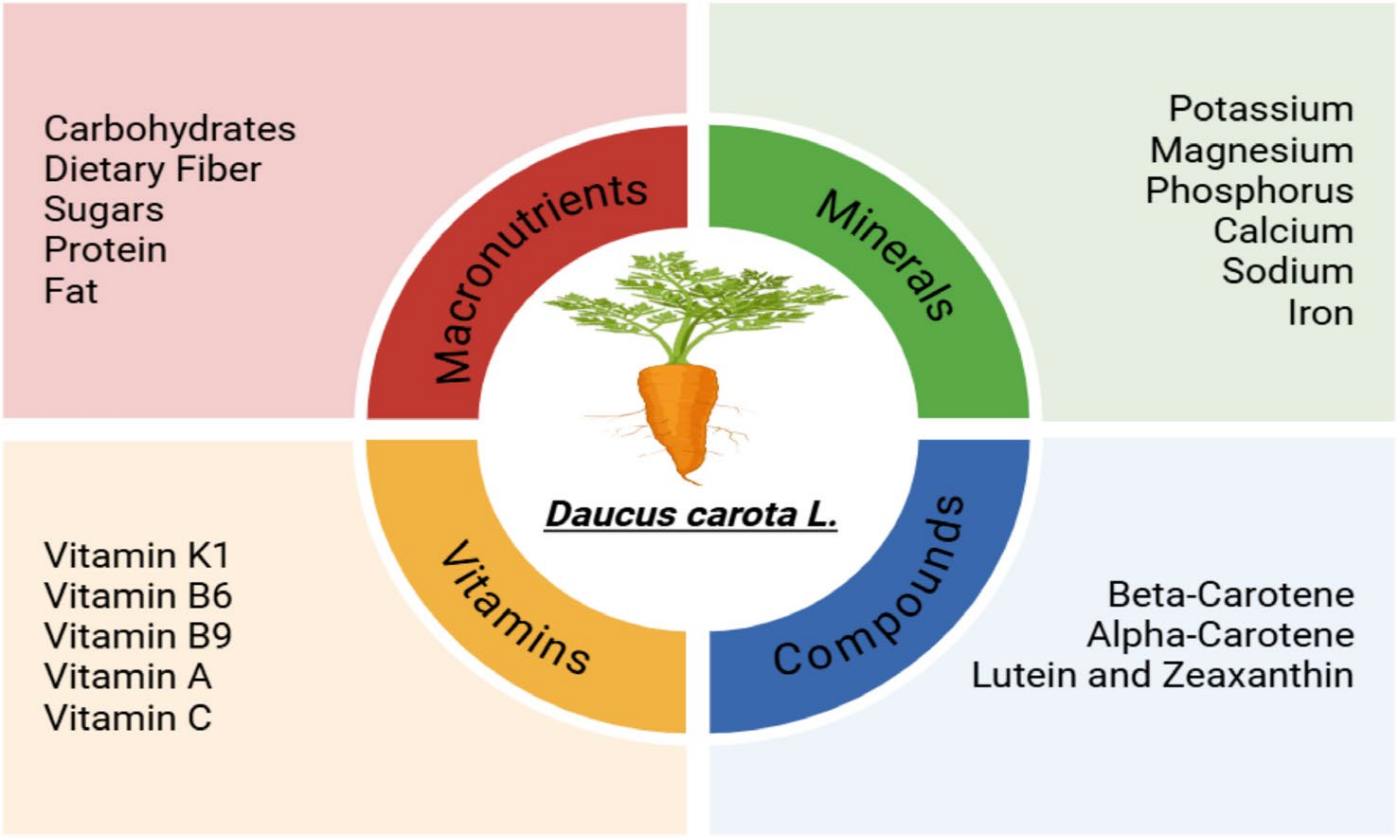
Nutritional components in carrot root.

Carrot root ( *Daucus carota* ) is rich in beta‐carotene, a provitamin A composite that is vital for healthy skin, good eyesight, and a healthy immune system (Yusuf et al. 2021). Beta‐carotene is tremendously antioxidant and orange in pigment. The carrot carotenoids are distinguished for their optimistic color (Koudela et al. 2021). Besides beta‐carotene, vitamins C and E complement beta‐carotene through the neutralization of free radicals and minimizing oxidative stress (Blando et al. 2021; Kim et al. 2023). Potassium, calcium, magnesium, and phosphorus are rich in carrots and are highly involved in various physiological processes ranging from enzyme operation to bone support and muscular contractions (Knez et al. 2022; Purewal et al. 2023). Carrots' dietary fiber stabilizes blood sugar levels and maintains healthy intestines (Schulzova et al. 2022). Apart from the nutritional value, the phenolic compounds in carrot root possess significant anti‐inflammatory and antioxidant properties (Figure 1) (Nguyen and Scarlett 2016). Mandrich et al. (2023) found that bioactive chemicals contribute to preventing chronic diseases such as heart disease and cancer. The bioactive chemical composition varies depending on carrot varieties and cultivation conditions. Their enhanced antioxidant activity with respect to orange carrots is explained by a series of environmental as well as genetic factors (Mizgier et al. 2016; Bhandari et al. 2022). The reason behind it is that black carrots contain lots of anthocyanins. Besides, organic acids and the sugars present in carrots make their taste better, as well as their utility in day‐to‐day life (Sharma et al. 2012). Carrots are chemically dense and are, therefore, a functional food with numerous beneficial influences on human health (Ding and Liu 2024; Que et al. 2019).

### 2.1 Bioactive and Phytochemical Profile of Carrots

**FIGURE 2:**
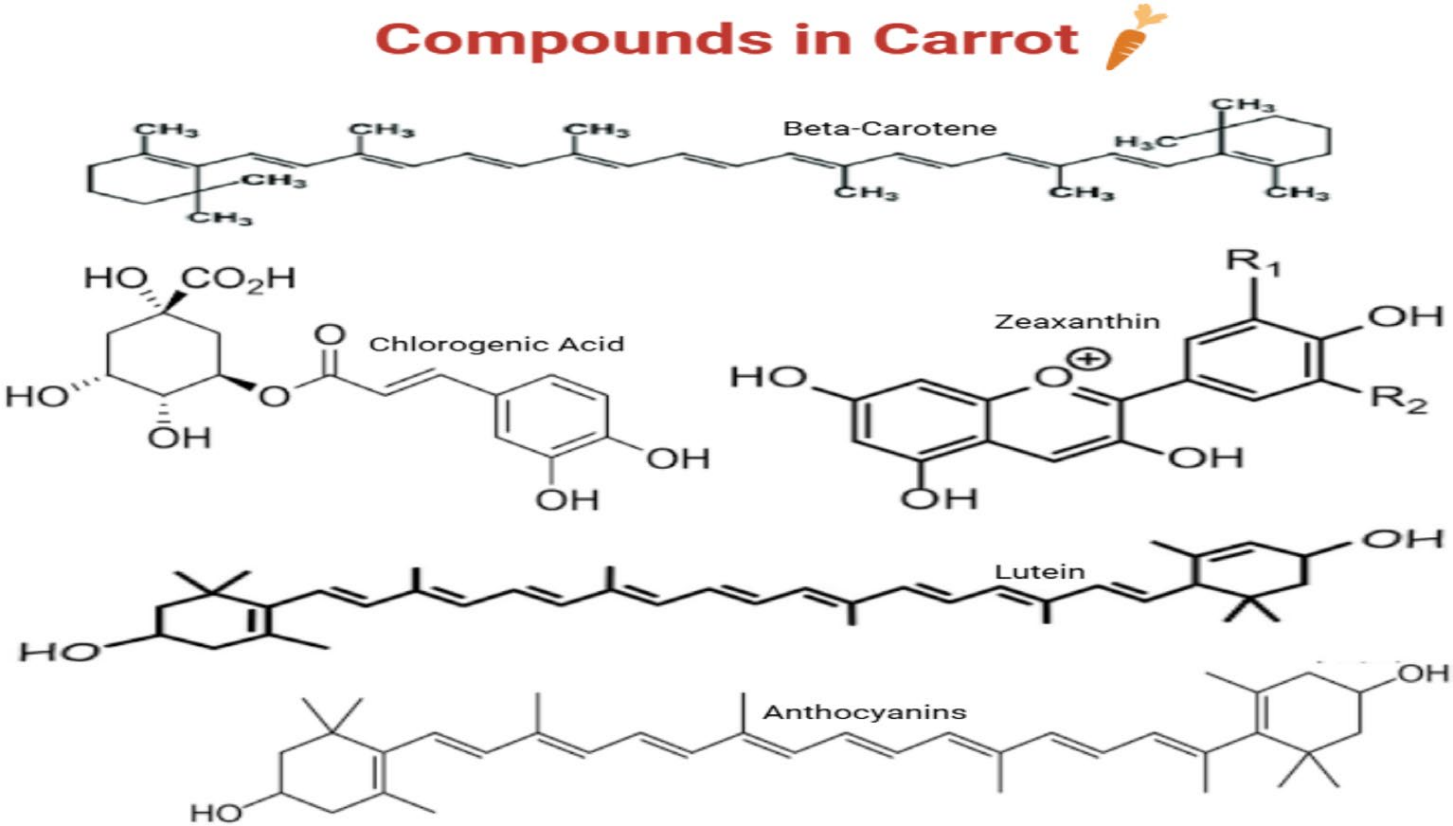
Structures of bioactive compounds in carrot.

Bioactive constituents such as carotenoids, flavonoids, and polyphenols are rich in carrot roots, enhancing their health benefits. Carrots are rich in antioxidants due to their polyphenols, including chlorogenic acid (Baidya and Sethy 2020; Roy et al. 2022). Oxidative stress is an established offender for immunological failure and chronic inflammation. Flavonoids constitute another type of chemical agent with immunomodulatory activities. They regulate inflammatory processes to enhance immunological function (Serafini and Peluso 2016; Safriani et al. 2022). The main beta‐carotene and other carotenoids are the source of carrots' health benefits (Figure 2). As per Fatima et al. (2020) and Uddin et al. (2023), these compounds are metabolized to vitamin A, which is essential for the development of immune cells and in preventing infections by sustaining epithelial barrier integrity. In addition to all that, carotenoids are antioxidants that reduce inflammation (Nasri et al. 2014; Ajenu et al. 2021). They accomplish this by reducing the production of inflammatory mediators and pursuing free radicals. Carrots have bioactive compounds that enhance immunity and reduce inflammation by various mechanisms. Polyphenols can help eliminate infection and suppress chronic inflammatory responses through the inhibition of nuclear factor‐kappa B (NF‐κB), a key proinflammatory cytokine regulator (Chang et al. 2025; Desjardins 2017; Kaur et al. 2023), such as tumor necrosis factor‐alpha (TNF‐α) and interleukin‐6 (IL‐6).

Flavonoids action on an immune cell procedure significant to adaptive immunity: the proliferation and instigation of T and B lymphocytes. Serafini and Peluso (2016) and Sindhu et al. (2019) exposed that these composites augment macrophage activity, which assists in reimbursing infections. This protection from inflated inflammation and immune responses is achieved by augmenting the innate immune mechanism and enhancing neutrophil and natural killer cell activities. These are also involved in producing anti‐inflammatory cytokines, such as interleukin‐10 (IL‐10), that aid in maintaining a healthy balance (Heena and Sunil 2019). As a functional foodstuff, carrots can improve immunity and decrease inflammation due to their bioactive composites. Because of this, these can contribute to the elevation of fitness as well as the management of prolonged diseases (Chauhan et al. 2020; Hosseini et al. 2018).

Carrot root, an enthusiastically obtainable vegetable, meaningfully subsidizes immune function because of its bioactive composites and robust nutritional profile. Ovando and González (2020) elucidate how it is valuable by citing its antioxidant capability, its function in vitamin A amalgamation, and its consequence on immune cell activation. Carotenoids such as beta‐carotene, lutein, and zeaxanthin are present in enormous amounts in carrot root and aid as antioxidants. Through the removal of free radicals, or extremely reactive molecules with the potential to abolish cellular structure, these antioxidants contest oxidative stress. The immune system may be diminished, making people vulnerable to infection and prolonged disease, as a consequence of this kind of oxidative impairment. The antioxidants found in carrots lessen oxidative stress, thereby augmenting the operation of the immune system (Baidya and Sethy 2020). Antioxidants also protect immune cells from oxidative stress, vital for their endurance and functionality throughout an immune response (Safriani et al. 2022). A decrease in inflammation and improved overall resilience of the immune system can be achieved through the addition of these dietary antioxidants, as per investigation (Serafini and Peluso 2016).

The provitamin A composite beta‐carotene is ironic in carrots. This subsidizes adaptive immunity conservation after the body adapts it to vitamin A. Moreover, defending the skin, mucous membranes, and epithelial tissues from contamination, vitamin A upholds the fitness of these tissues (Serafini and Peluso 2016). Important immune system elements T and B lymphocytes are also profoundly impacted by it throughout their progress and differentiation. Through the process of distinguishing and abolishing pathogenic microbes, such cells play an operative function in immunization (Bahrami et al. 2018). This is where the significance of carrot form beta‐carotene is additionally obvious since appropriate vitamin A altitudes are complicated in modifying immunoglobulin amalgamation, proteins that are vigorous to humoral immunity (Hosseini et al. 2018).

To control the immune system, phytochemicals from carrot root act on the activity of immune cells and cytokine fabrication. The immune reaction is contingent on a harmonious equilibrium that comprises the fabrication of cytokines, which are signaling fragments. A vigorous immune system hinges on an incessant source of lymphocytes and macrophages, both of which carrots offer plentifully (Akbari et al. 2022). Besides regulating inflammatory responses, the substances promote the amalgamation of anti‐inflammatory cytokines; these cytokines are essential for averting prolonged inflammation but can negotiate immunity (Hosseini et al. 2018). Given these interpretations, carrots can be exploited as an immunomodulatory foodstuff (Safriani et al. 2022). Carrot root is auspicious to augment immunological roles because of its antioxidant properties, its purpose in vitamin A construction, and its effect on cytokine fabrication and immune cell function. Carrots contain a high concentration of bioactive compounds that enhance immune function's ability to resist and recover from a vast array of diseases. As a demonstration of how functional foods play a role in general well‐being, reflect upon how easy and effortless it would be to enhance immunity by consuming more carrots.

## 3 Papaya: Nutrient Profile

**FIGURE 3:**
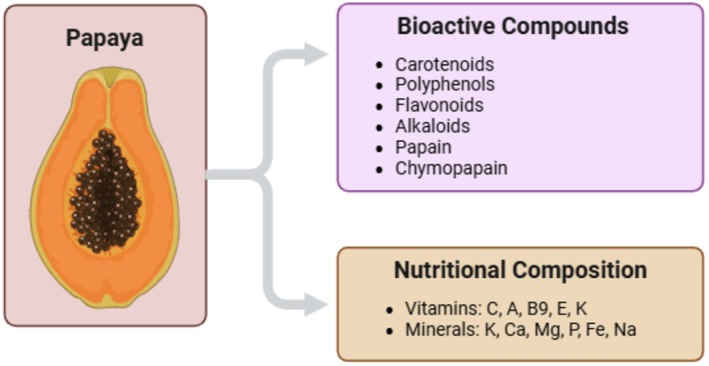
Bioactive compounds and nutritional composition of papaya.

Vitamins A, C, and E are some of the bioactive compounds in the nutrient‐dense *Carica papaya* fruit that is very highly valued for nutritional purposes (Figure 3). Khor et al. (2021) and Agada et al. (2021) reported that these compounds are endowed with powerful antioxidant, anti‐inflammatory, and immunomodulatory activities. Papayas contain a high content of vitamin A that assists the body's first defense against infection, the mucosal tissues, in staying intact and functioning effectively. Oche et al. (2017) and Ovando‐Martinez et al. (2018) posit that it aids in the well‐being of the immune system. Vitamin C and other robust antioxidants assist the immune system to create and employ more efficient cells such as lymphocytes and neutrophils (Jeon et al. 2022; Pierson et al. 2012). Annegowda and Bhat (2016) and Laurora et al. (2021) identified that vitamin E defends the cell membrane from oxidative deterioration, modifies immunological reactions, and impacts T‐cell functionality. Soib et al. (2020) and Palanisamy and Basalingappa (2020) discovered that papaya extracts are rich in the proteolytic papain enzyme that has therapeutic properties such as reducing inflammation and enhancing wound healing. Papain improves immunological wellness by improving the bioavailability of nutrients, as well as restraining the generation of inflammatory developments within proteins, conferring to Nguyen et al. (2016) and Niveditha et al. (2020). Uribe et al. (2015) and Abdel‐Halim et al. (2021) recognized that papaya's conformation renders it a functional foodstuff with the competence of dropping inflammation, improving immunological maintenance, and providing antioxidant protection.

### 3.1 Bioactive Profile and Phytochemicals in Papaya

Papaya comprises a multitude of bioactive composites like alkaloids, flavonoids, and lycopene that deliberate on its medicinal assistance. The leaves, seeds, and fruit of the plant have chemical composites that have antioxidant, immunomodulatory, and anti‐inflammatory action. Lycopene is a carotenoid that mainly occurs in the fruit pulp. This is a powerful antioxidant that traps free radicals and acts in contradiction to oxidative stress. Its activity contrary to inflammation and the improvement of the immune reaction is well recognized. With respect to inflammatory indicators such as cytokines, lycopene has been related to reduced levels, which designates that it might be operative in anti‐inflammatory illnesses (Khor et al. 2021). Cardiovascular illness, cancers, and other prolonged oxidative impairment illnesses can be prohibited by its support (Agada et al. 2021; Laurora et al. 2021). Papaya flavonoids establish another important category of bioactive substances with abundant optimistic health allegations. Oche et al. (2017) and Jeon et al. (2022) present that polyphenolic elements in papaya seeds and leaves have effective anti‐inflammatory properties by hindering cyclooxygenase and lipoxygenase enzymes. Flavonoids are beneficial in the contest against inflammation and infections due to their capability to regulate immune cell functions, decrease oxidative stress, and alleviate cell membranes (Uribe et al. 2015; Nguyen et al. 2016).

Others, like carpaine, ensue in papaya's leaves and seeds and own strong anti‐inflammatory and antibacterial actions. To accomplish immune‐arbitrated illnesses, papaya alkaloids act by assimilating cellular paths to decrease inflammation and oxidative stress (Abdel‐Halim et al. 2021). Besides, inflection of immunological paths improves the body's immunity in contradiction to pathogens (Nguyen et al. 2016; Sindhu et al. 2019). The anti‐inflammatory properties have been accredited to the varied range of bioactive and antioxidant compounds in papaya extract. These prevent the formation of cytokines, which are involved in the inflammatory process, for example, interleukin‐6 (IL‐6) and tumor necrosis factor‐alpha (TNF‐α) (Jeon et al. 2022). Studies have established that papaya leaf extracts have the potential to inhibit inflammation in animal models; hence, they are likely to be applied in the treatment of chronic inflammatory diseases, inflammatory bowel disease, and arthritis (Soib et al. 2020; Vega‐Gálvez et al. 2019). The most outstanding aspect of flavonoids' anti‐inflammatory properties is their hitting several inflammatory pathways. Flavonoids relieve inflammation‐intensive conditions by downregulating the production of inflammatory mediators by blocking the activity of NF‐κB (Nguyen et al. 2016; Roy et al. 2022).

Papaya extracts also act as a preservative of immunological activity. Alkaloids and flavonoids have exhibited bioactivity in innate and adaptive immune responses. Increments in phagocytic activity and immunological surveillance efficacy of papaya alkaloids demonstrate improved body defenses against infections (Nguyen et al. 2016; Wani and Uppaluri 2022). By modulating the function of immune cells such as T lymphocytes and macrophages and reducing oxidative stress that may impair the immunity function, flavonoids have been proven to support immunomodulation (Agada et al. 2021; Safriani et al. 2022).

Furthermore, Niveditha et al. (2020) examined in an investigation that papaya leaf extracts can possibly increase the immune system by growing platelet counts and immunological function in patients anguishing from dengue fever. Also, lycopene defends immune cells from free radical injury and upholds immunity at its peak throughout an immune reaction (Vega‐Gálvez et al. 2019). Papaya extracts comprise greater levels of lycopene, flavonoids, and alkaloids that contribute to their powerful anti‐inflammatory and immunomodulatory actions. Generally, these composites decrease oxidative stress, affect inflammatory pathways, and improve the immune system. Because of this, it can be used for immune system maintenance and prolonged inflammatory illnesses as a natural medication.

Their usage in modern medication will upsurge as investigators inspect their bioavailability and more effective approaches to extraction. Among these, the immunomodulatory actions of papaya extracts prepared from numerous portions of the papaya plant ( *Carica papaya* ) have been gaining specific consideration. These advantages are based on enzyme content, antioxidant capacity, and bioactive substances affecting cytokine balance, T‐cell activation, and innate immunity. The proteolytic enzyme papain, occurring in papayas, breaks down proteins into peptides to increase nutritional uptake. Improved digestion helps the immune system by guaranteeing the body has all the nutrients necessary for effective immunological responses (Husen and Iqbal 2021). The fruit and leaves, in addition, have high contents of antioxidants that fight oxidative stress, such as vitamin C, carotenoids, and flavonoids. Antioxidants protect the immune cells from oxidative stress; this affects and damages cells and tissues and ultimately distorts immunological functions (Jantan et al. 2015; Weyh et al. 2022).

Papaya has a great deal of vitamin C, which enhances the action of neutrophils and natural killer cells, two essential components of innate immunity. Highly antioxidant‐rich diets, like papayas, are critical for maintaining immunological well‐being because these cells provide the first set of defenses against pathogens within the body (Jiang et al. 2021). An additional way papayas endorse immunological homeostasis is through counteracting reactive oxygen species (ROS).

Di Sotto et al. (2020) exposed that when inflammation reduces, immunological stability is improved. Papaya extracts affect adaptive immunity by modifying cytokine amalgamation and inspiring T cells. Alkaloids, flavonoids, and phenolics, which are bioactive composites in papaya, persuade T‐cell proliferation and differentiation, an acute step in the fabrication of immune reactions intended at definite pathogens (Baraya et al. 2017).

Furthermore, papaya controls the equilibrium of cytokines that persuade inflammation to those that overwhelm it. Investigation studies have revealed that cytokine fabrication, such as tumor necrosis factor‐alpha (TNF‐α) and interleukin‐10 (IL‐10), was moderated by papaya leaf extracts. Nguyen et al. (2016) specified that this regulation assists in overwhelming pathogenic inflammation by supporting immune enthusiasm. As per Husen and Iqbal (2021), a steadiness cytokine reaction can avert chronic inflammation, immunological destruction, and a diversity of disorders. Numerous studies have established that papaya has immune‐boosting properties. Improved macrophage function and cytokine amalgamation are indications of the potential of papaya leaf extracts to exhibit boosting effects by both innate and adaptive immunity (Olayem et al. 2024). Papaya supplementation improved the activity of antioxidant enzymes in individuals and thereby vindicated its function in protecting immune cells against impairment inflicted by oxidative processes, as per diverse investigations (Di Sotto et al. 2020).

Moreover, papaya extract hastens healing and improves the immune system reaction in clinical infections. In dengue fever, for instance, investigators have newly emphasized bioactive values of the seed and leaves of the papaya that grasp promise in plummeting viral loads with improved induction of platelet amalgamation and immunological role (Alhazmi et al. 2021). Because of their enzymatic activity, antioxidant capability, and capacity to regulate T‐cell function and steadiness of cytokines, the papaya extracts suggest numerous assistances associated with fitness in immunology. These characteristics make papaya a perfect food to add to your diet if you would like to improve your immune system, both natural and adaptive. Plant‐functional foods such as papaya are excellent for your health and immune system, based on the given data set. The content of carrot root and papaya extract bioactive components is presented in Table 1.

**TABLE 1:** Composition and bioactive compounds of carrot root and papaya extracts and their role in immunity and inflammation.

Aspect	Carrot root	Papaya extracts
Key nutrients	Beta‐carotene (provitamin A), vitamins A, C, K, B6, potassium, magnesium, calcium, and dietary fiber (Sharma et al. 2012; Yusuf et al. 2021)	Vitamins A, C, E, folate, potassium, magnesium, and calcium. Contains papain, chymopapain, and fiber (Agada et al. 2021; Palanisamy and Basalingappa 2020)
Primary bioactive compounds	Polyphenols (e.g., chlorogenic acid, caffeic acid), flavonoids (quercetin, luteolin), and carotenoids (beta‐carotene, alpha‐carotene, lutein) (Que et al. 2019; Mizgier et al. 2016)	Lycopene, flavonoids (quercetin, kaempferol), alkaloids, and phytosterols. Also contains bioactive enzymes like papain (Jeon et al. 2022; Wani and Uppaluri 2022)
Antioxidant activity	High due to carotenoids and polyphenols. Neutralizes reactive oxygen species (ROS), reducing oxidative damage (Schulzova et al. 2022; Sharma et al. 2012)	Papain and flavonoids neutralize ROS and improve cellular antioxidant defenses. Lycopene is a potent antioxidant (Vega‐Gálvez et al. 2019; Palanisamy and Basalingappa 2020)
Immune system support	Beta‐carotene enhances T‐cell activity and improves mucosal immunity. Polyphenols boost lymphocyte function (Calder et al. 2020; Yusuf et al. 2021)	Vitamin C promotes white blood cell production. Papain supports antigen digestion, enhancing immune surveillance (Childs et al. 2019; Pathak et al. 2022)
Anti‐inflammatory pathways	Inhibits proinflammatory mediators like TNF‐α, IL‐6, and COX‐2. Modulates NF‐κB signaling, reducing inflammation (Mizgier et al. 2016; Nguyen et al. 2016)	Suppresses inflammatory cytokines, including IL‐1β and prostaglandins. Modulates COX‐1 and COX‐2 activity through flavonoids (Jeon et al. 2022; Laurora et al. 2021)
Gastrointestinal benefits	Dietary fiber improves gut microbiota composition, supporting immune health. Carotenoids protect the intestinal mucosa (Weyh et al. 2022; Yusuf et al. 2021)	Papain improves protein digestion and reduces intestinal inflammation. Supports gut barrier integrity (Nguyen et al. 2016; Pathak et al. 2022)
Skin health	Beta‐carotene and vitamin C promote collagen production and protect against UV‐induced oxidative stress (Mandrich et al. 2023; Mizgier et al. 2016)	Vitamin C and E enhance wound healing. Papain supports cell regeneration and reduces scars (Soib et al. 2020; Palanisamy and Basalingappa 2020)
Cardiovascular health	Potassium helps regulate blood pressure, while carotenoids reduce LDL oxidation (Knez et al. 2022; Yusuf et al. 2021)	Lycopene and flavonoids improve vascular function and reduce oxidative stress in arteries (Jeon et al. 2022; Vega‐Gálvez et al. 2019)
Anticancer potential	Polyphenols and carotenoids inhibit tumor growth through ROS modulation and apoptotic pathways (Yusuf et al. 2021; Que et al. 2019)	Flavonoids and alkaloids suppress tumor proliferation by inhibiting key signaling pathways like NF‐κB (Nguyen et al. 2016; Jeon et al. 2022)
Enzyme‐related effects	Enzymes in carrots (e.g., peroxidase) assist in detoxification and reduce inflammatory markers (Mizgier et al. 2016; Sharma et al. 2012)	Papain aids in reducing systemic inflammation by breaking down complex proteins and antigens (Nguyen et al. 2016; Pathak et al. 2022)
Metabolic benefits	Improves insulin sensitivity and reduces inflammation linked to metabolic syndrome (Nguyen et al. 2016; Yusuf et al. 2021)	Modulates lipid metabolism and improves glycemic control. Papain assists in managing metabolic inflammation (Vega‐Gálvez et al. 2019; Palanisamy and Basalingappa 2020)

## 4 Role of Carrot Root and Papaya Extract in Combating Inflammation

**FIGURE 4:**
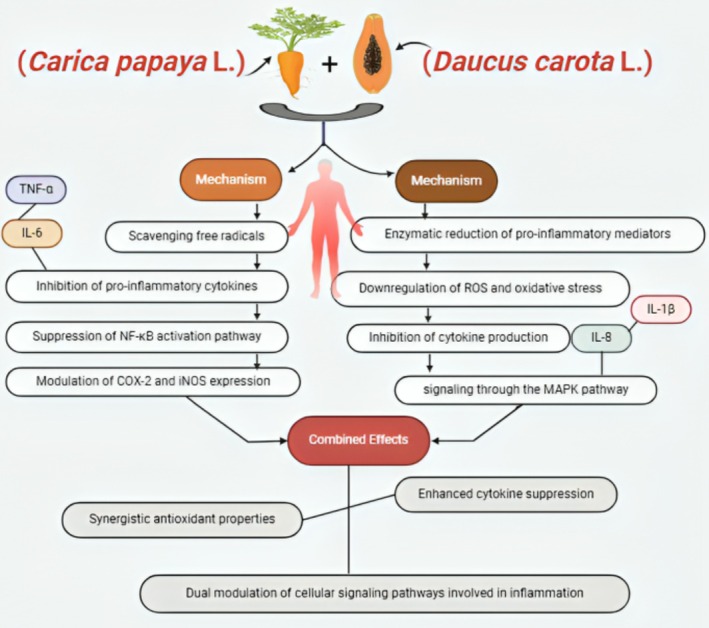
Mechanism of actions; carrot root and papaya.

Based on a recent report by Carpena et al. (2022), carrots, especially their roots, possess anti‐inflammatory properties as they are laden with bioactive chemicals like carotenoids and polyphenols. Both oxidative stress and inflammatory reactions lead to medical problems associated with inflammation, and these chemicals are needed to reduce both (Figure 4). As a result of the antioxidant activity and effect on several molecular pathways regulating inflammation, the bioactive compounds in carrot roots render them a potential therapeutic diet for inflammation‐associated diseases. The anti‐inflammatory effect of carrot root is presented in Table 2.

**TABLE 2:** Anti‐inflammatory role of carrot root.

Mechanism	Description	References
Anti‐inflammatory mechanisms of carotenoids and polyphenols	Carrot root contains carotenoids (β‐carotene, lutein) and polyphenols, which neutralize free radicals and inhibit inflammatory enzymes like COX‐2	Yusuf et al. (2021), Mandrich et al. (2023), Sharma et al. (2012)
Reduction of proinflammatory cytokines	Carrot extracts downregulate the expression of proinflammatory cytokines such as TNF‐α and IL‐6 in vitro, reducing inflammation	Mizgier et al. (2016), Childs et al. (2019)
Effects on oxidative stress and inflammatory pathways	Antioxidants in carrots reduce oxidative stress, inhibit NF‐κB activation, and suppress the release of ROS, preventing chronic inflammation	Nguyen and Scarlett (2016), Schulzova et al. (2022), Calder et al. (2020)
Inhibition of COX and LOX enzymes	Polyphenols in carrot root suppress the activity of cyclooxygenase (COX) and lipoxygenase (LOX), enzymes critical in the synthesis of inflammatory mediators like prostaglandins	Kim et al. (2023), Shahidi and Ambigaipalan (2015)
Promotion of anti‐inflammatory cytokines	Carrot compounds increase the production of anti‐inflammatory cytokines such as IL‐10, enhancing immune regulation	Patil et al. (2021), Kobaek‐Larsen et al. (2023)
Gut microbiota modulation	Dietary fibers in carrots support gut health by promoting beneficial microbiota, which indirectly regulate systemic inflammation	Slavin and Lloyd (2012), Xiang et al. (2021)

### 4.1 Anti‐Inflammatory Activity: Carotenoids and Polyphenols of Carrot Root

Carrots are an excellent source of vitamin A because they contain high concentrations of carotenoids, especially β‐carotene, which the body can convert into vitamin A. To avoid initiating and sustaining inflammatory reactions, carotenoids such as lutein and α‐carotene trap free radicals and inhibit the formation of reactive oxygen species (ROS) (Nguyen and Scarlett 2016). It lowers oxidative stress, a prime factor in chronic inflammation, and mops up free radicals, according to Blando et al. (2021). Carotenoids blunt the inflammatory signaling pathways directly by lowering the activity of nuclear factor‐kappa B (NF‐κB), a principal mediator of inflammation (Schulzova et al. 2022). Aside from its anti‐inflammatory action, carrots have polyphenols, which consist of flavonoids and phenolic acids. Two among the numerous pathways through which such polyphenolic compounds diminish inflammation are by inhibiting cyclooxygenase (COX) enzymes and the regulation of cytokine production (Yusuf et al. 2021). Besides, polyphenols can stimulate the Nrf2 pathway, which enhances antioxidant defenses and decreases inflammatory cytokine levels (Blando et al. 2021). The anti‐inflammatory properties of carotenoids and polyphenols are demonstrated by these mechanisms.

Carrot root extracts have been exposed to decrease proinflammatory cytokine levels like interleukin‐6 (IL‐6) and tumor necrosis factor‐alpha (TNF‐α), as per investigations. Agarwal and Shanmugam (2022) recognized that chronic inflammatory illnesses like arthritis and heart illness are frequently characterized by augmented levels of these cytokines, which are intricate in inflammation. Experts exposed bioactive composites in carrot roots that have anti‐inflammatory effects by overwhelming the expression of certain cytokines (Luo et al. 2024). For occurrence, numerous in vitro and in vivo investigations recognized that β‐carotene suggestively inhibits TNF‐α and IL‐6 fabrication (Kim et al. 2023). As investigated by Yusuf et al. (2021), β‐carotene ironic extracts from carrot root hinder the activation of NF‐κB, a transcription factor that produces proinflammatory cytokines. Inflammation is reduced because of the deterioration in the activity of NF‐κB, thus decreasing the production of TNF‐α and IL‐6. Schulzova et al. (2022) explain that carrot roots have flavonoids and phenolic acids, which affect immune cells such as macrophages and T lymphocytes. Consequently, fewer cytokines are released by these cells. The treatment of inflammation‐related diseases is based on its ability to downregulate inflammatory cytokines, and it naturally has this ability, thus making it an option to anti‐inflammatory drugs (Zhang et al. 2024). Carrot root's antioxidant function is the main way through which it alleviates inflammation. Carotenoids and polyphenols in carrots, according to Sibeko et al. (2021), reduce oxidative stress, a key trigger of the inflammatory cascade.

A reactive oxygen species (ROS) imbalance and antioxidant defenses induce oxidative stress. Both inflammation responses and cell injury are induced by oxidative stress (Blando et al. 2021). Oxidative stress induces the NF‐κB pathway, which is one of the principal pathways for inflammation. Nguyen and Scarlett (2016) reported that carotenoids, including β‐carotene, inhibit proinflammatory gene activation by inhibiting the NF‐κB signaling pathway. As per Kim et al. (2023), NF‐κB suppression restricts inflammatory mediator production, including cytokines, chemokines, and adhesion molecules. These molecules facilitate the recruitment of immune cells to inflamed tissues. The carrot root constituents also reduce oxidative stress levels by scavenging free radicals and increasing in vivo antioxidant defense mechanisms like superoxide dismutase (SOD) and catalase (Agarwal and Shanmugam 2022).

The anti‐inflammatory effects of the carrot root extracts are enhanced more by their scavenge mechanism of ROS coupled with modification of antioxidant defenses. A large amount of carrot root carotenoids and polyphenols dramatically reduce inflammation. These substances have anti‐inflammatory actions because they lower oxidative stress, regulate cytokine production, and inhibit critical inflammatory pathways through NF‐κB blocking. The natural ingredients of carrots become a bioactive reservoir that can help fight chronic inflammation, one of the root causes of many illnesses, such as metabolic disorders, cardiovascular diseases, and arthritis. Carrot root serves as a valuable nutritional component to enhance health and, not least, to lessen inflammation‐related disorders, even if the processes by which carrots exert their anti‐inflammatory effect remain yet to be researched.

### 4.2 Anti‐Inflammatory Activity of Papaya Extracts

**FIGURE 5:**
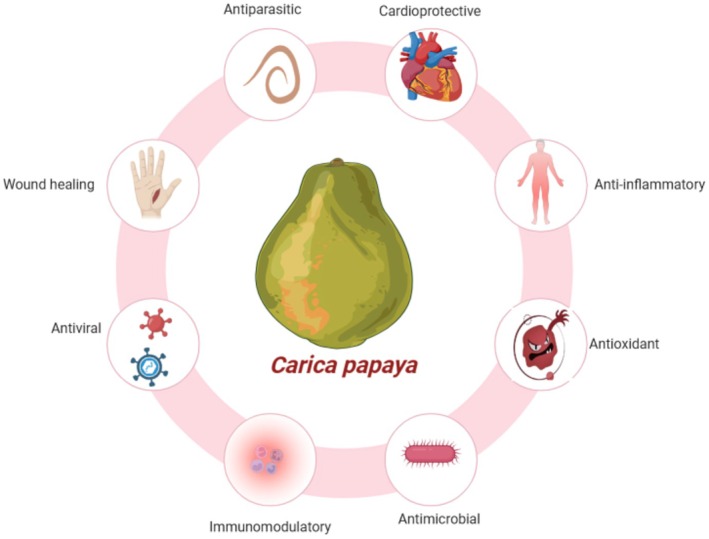
Medicinal properties of papaya.

It has been established for a long time that papayas, that is, *Carica papaya* , possess medicinal benefits, mainly in reducing inflammation (Figure 5) (Biswas et al. 2023). These include the results from the bioactive compounds of papaya, like phenolic compounds and other antioxidants, as well as its enzymes, such as papain. Research on the anti‐inflammatory properties of papaya on both acute and chronic inflammatory diseases demonstrated an alteration of several inflammation pathways. Papain has proteolytic enzyme properties isolated from papaya fruit and leaves. Powerful anti‐inflammatory properties have been examined in it, mainly working on breaking down proteins involved in the inflammatory response (Anal et al. 2023). Investigation indicates that papain is able to reduce pain and edema in tissues by acting on inflammatory mediators (Jeon et al. 2022). By degrading inflammatory proteins and decreasing the concentration of proinflammatory cytokines, it inhibits the inflammatory process. Other anti‐inflammatory activities of papaya are due to other enzymes like chymopapain. These enzymes are capable of modulating the immune system based on their action on neutrophils, macrophages, and other key immune cells that are engaged in inflammation. Papayas contain enzymes like papain, which facilitate the degradation of proinflammatory proteins, thereby reducing inflammation systemically and locally (Soib et al. 2020). The papaya extracts' enzymatic actions have inferences on the likelihood of it being a medication for disease procedures that comprise unchecked inflammation. Inflammation indicators in both prolonged and acute inflammatory illnesses are, as designated by Majeed and Bhat (2022), reduced with the usage of papaya extracts.

There is a certain indication that papaya enzyme extracts decrease acute inflammation, which is noticeable in redness, swelling, and pain. This is described by Alhazmi et al. (2021) as this effect being established in animal prototypes by decreasing important indicators of inflammation such as TNF‐α and CRP. In contradiction to this context, papaya extracts can be of abundant prospective as a therapeutic intervention for acute inflammatory circumstances, such as burns, contaminations, and trauma. Prolonged inflammatory indicators are related to autoimmune illnesses, cardiovascular disease, arthritis, and other prolonged diseases, though papaya extracts are proficient in reducing these indicators Deis et al. (2021). Investigations have designated that the concentrations of the proinflammatory cytokines TNF‐α and interleukin‐6 (IL‐6) are abridged when papaya extract is consumed. These two cytokines play a noteworthy role in the sustenance of prolonged inflammatory illnesses Jeon et al. (2022).

Moreover, the antioxidant exploite of papaya decreases oxidative stress, a predominant reason for prolonged inflammation Jeon et al. (2022). Because of its capability to change the indicators of acute and prolonged inflammation, papaya extracts are a multilayered treatment. Table 3 specifics how papaya extract affects inflammation.

**TABLE 3:** Anti‐inflammatory role of papaya extracts.

Mechanism	Description	References
Anti‐inflammatory activity of papain and other enzymes	Papain, a proteolytic enzyme in papaya, degrades inflammatory mediators, and inhibits immune overactivation	Jeon et al. (2022), Laurora et al. (2021), Al‐Seadi et al. (2021)
Reduction of inflammation markers	Papaya leaf and seed extracts lower inflammation markers like CRP, TNF‐α, and IL‐6 in acute and chronic inflammation	Agada et al. (2021), Uribe et al. (2015)
Modulation of inflammatory signaling pathways	Compounds in papaya (e.g., flavonoids, alkaloids) regulate inflammatory signaling cascades such as MAPK and NF‐κB, reducing systemic inflammation	Cao et al. (2024), Arulselvan et al. (2016)
Antioxidant and anti‐inflammatory synergy	Papaya contains vitamins C and E, and phenolic compounds that act synergistically to combat oxidative stress, thereby reducing inflammation	Jeon et al. (2022), Shaban et al. (2021)
Reduction of edema and swelling	Papaya enzyme extracts show efficacy in reducing localized edema and swelling in experimental models, attributed to enzyme‐mediated fibrinolysis	Yang et al. (2023), Jimoh‐Abdulghaffaar et al. (2023)
Improved wound healing with anti‐inflammatory effects	Topical application of papaya extract accelerates wound healing by reducing local inflammation and promoting tissue regeneration	Marlinawati et al. (2023), Vijayakumar et al. (2024)
Immunomodulatory benefits	Polysaccharides enhance macrophage activity and regulate lymphocyte function, providing a balanced immune response that avoids chronic inflammation	Cao et al. (2024), Astuti et al. (2020)

#### 4.2.1 Modulation of Inflammatory Signaling Pathways

NF‐κB, the principal regulator of the inflammatory reaction, is precisely targeted by the papaya extracts on numerous crucial signaling paths. NF‐κB activation results in prolonged inflammation and leads to the production of numerous cytokines and mediators that can be a cause of inflammation. The initiation of proinflammatory mediators such as TNF‐α, IL‐1, and IL‐6 is reduced by employing papaya extracts rich in papain and other antioxidants as a suppressor of NF‐κB activation (Alhazmi et al. 2021). Afterwards, it disrupts the inflammatory processes leading to tissue damage and prolonged inflammatory illnesses. NF‐κB activation is crucial for the conquest to accomplish inflammation.

Furthermore, papaya regulates inflammation through interfering with the mitogen‐activated protein kinase (MAPK) signaling path. Concerning stress and inflammation, the MAPK signaling path plays an important function in cell signaling. Grounded on an investigation by Jeon et al. (2022), papaya extracts can be rummaged to constrain the activation of this path, resulting in fewer inflammatory indications.

Separately from dropping inflammation and protection cells in contradiction to oxidative stress, papaya extracts trigger antioxidant‐indicating paths like the Nrf2 path (Soib et al. 2020). Papaya extracts own broad‐spectrum anti‐inflammatory action as a consequence of their mutual effect on the NF‐κB and MAPK paths. Papaya extracts, particularly papain and other enzyme fractions, own strong anti‐inflammatory activity. In both prolonged and acute disorders, this is because of the fact that it has a dropping effect on important inflammation markers, including TNF‐α and IL‐6 (Jeon et al. 2022). Significant pathways for the inflammatory procedure, such as MAPK and NF‐κB, are exaggerated by papaya. These actions designate that papaya extracts own the capability to cure a diversity of inflammatory sicknesses clearly, therefore interpreting them one of the most important plants associated treatments in contemporary medication.

## 5 Synergistic Effects When Combining Carrot Root and Papaya Extracts

Carotenoids, polyphenols, enzymes, and other bioactive composites found in carrot and papaya root extracts comprise strong antioxidant and anti‐inflammatory properties (Santana Gálvez 2020). According to Prasad et al. (2017), these extracts can interact with each other in such a way that it improves the therapeutic potential of both of them. Aside from anti‐inflammatory properties, the carotenoids in carrot roots such as beta‐carotene utilize strong antioxidant activities that abolish free radicals and oxidative stress (Chauhan et al. 2020). Additionally, validate that papaya extract, particularly the papain proteolytic enzyme, moderates immune reactions and damages inflammatory proteins. By reducing oxidative stress and generating inflammatory cytokines by multiple mechanisms, such drugs can likely work together to reduce the body's overall inflammatory burden. By pointing out unique phases of the inflammatory process, a combination of papaya and carrot root extracts can improve the potential of managing chronic inflammatory diseases such as arthritis and cardiovascular disease (Akbari et al. 2022).

### 5.1 Application in Functional Foods and Nutraceuticals

Amalgamation of papaya and carrot root extracts has excessive potential for the advancement of nutraceuticals and functional mealtimes. The nutritional benefits of both plants are already known; papaya is rich in vitamins, antioxidants, and enzymes such as papain, while carrots have an abundance of vitamins, particularly vitamin A (in the form of beta‐carotene) (Ajenu et al. 2021). Because of their anti‐inflammatory, antioxidant, and immune‐boosting properties, including these extracts in functional foods may further enhance the health benefits achieved through conventional diets. For instance, carrot and papaya extracts can be added to salads, smoothies, or even snacks for general improvement in immune function and reduction in inflammation (Kafuko 2021).

The preparation may be used in various forms, such as beverages, powders, or capsules for nutraceutical drug formulations in inflammatory and oxidative stress cases. In specific conditions such as inflammation, oxidative stress, and prevention of chronic diseases, its synergistic activity would likely lead to a well‐rounded strategy in disease prevention and management (Nasri et al. 2014). Moreover, their blend of nutraceuticals could fulfill the mounting demand for plant‐based, natural supplements that offer valuable benefits without the adverse effects sometimes associated with pharmaceutical drugs (Khan et al. 2023).

### 5.2 Role in Dietary Strategies to Manage Immune and Inflammatory Disorders

Due to a greater concern for their health, dietary therapies for inflammatory and immunological diseases have received more attention as part of a more holistic approach to health. Both papaya and carrot root extracts contain anti‐inflammatory properties, making them excellent options for such strategies (Liang et al. 2023; Khodaie et al. 2024). Chronic inflammation often accompanies many autoimmune diseases, metabolic disorders, and cancer (Hosseini et al. 2018). A diet rich in fruits and vegetables, including extracts from papaya and carrots, can reduce inflammation by regulating immune responses and lowering the synthesis of proinflammatory cytokines (Cicio et al. 2022). Carrot carotenoids are not only antioxidants but also have immunomodulatory properties, which enhance the capability of an infection, fight it, and reduce exaggerated inflammatory reactions (Roy et al. 2022).

Papaya papain enzymes dissolve proteins that cause inflammatory reactions at the cellular level, thereby decreasing inflammation (Bahrami et al. 2018). As they simultaneously deal with the immune system and inflammation, it is possible to plan a diet to treat diseases such as rheumatoid arthritis, asthma, or even inflammatory bowel disease (IBD) (Ahuja et al. 2021). Incorporation of papaya and carrot extracts into the diet through whole foods and supplements may be more beneficial, especially for those who wish to combat inflammation and boost their immune systems without the use of prescription drugs (Holkem et al. 2023). As perceived by the increasing marketplace of functional foodstuffs, such dietary methods conform with the progressively natural and preventive method to health tendencies between customers (Hosseini et al. 2018).

## 6 Evidence From Preclinical and Clinical Studies

### 6.1 Vitro and Vivo Studies; Carrot and Papaya Extracts

Preclinical investigations established that papaya and carrot extracts controlled the immune system and repressed inflammation. Kure et al. (2017) investigation described that carrot root extracts, which comprise beta‐carotene and other carotenoids, had prospective anti‐inflammatory activity in animal experimentations. Bahrami et al. (2018) in their investigation described that carrot extract improved immunological activity and abridged inflammatory indicators in mice subjected to chemically induced inflammation. Based on Chauhan et al. (2020), in vitro trials have exposed that carrot extract overwhelms the activation of NF‐κB, a significant pathway complicated in inflammation, representing that it can moderate the inflammatory reaction and overwhelm cytokine fabrication.

Additionally, animal investigation on papaya extracts has also revealed promising effects in decreasing inflammation levels and immune reactions. Sindhu et al. (2019) detected that arthritic rats getting papaya seed extract had meaningfully lesser proinflammatory cytokine levels (TNF‐α, IL‐6), representing that it has an anti‐inflammatory prospective. Additionally, in vitro experimentations have established that papain, a plant proteolytic enzyme mined from papaya, is proficient in altering how immune cells persuade an inflammatory reaction by constraining their fabrication of main inflammatory mediators.

The prospective health effects of carrot and papaya extracts have been the emphasis of numerous clinical investigations, where these have been inspected for their capability to inhibit inflammation and disturb the immune system. Hosseini et al. (2018) exposed those persons suffering from prolonged inflammatory diseases who spent carotenoids from carrots in their diets and had meaningfully lesser blood levels of C‐reactive protein (CRP) (Li et al. 2025). This recommends that the carrot root can deliver systemic inflammation regulation for individuals. Clinical studies on papaya extracts have also shown promising results in curing inflammation‐related diseases. For example, clinical research on the benefits of the extract of papaya leaves on rheumatoid arthritis patients established that it brought substantial relief in the case of inflammation and pain accompanied by an improvement in joint functionality (Cicio et al. 2022). Such clinical results validate the anti‐inflammatory properties of extracts from papaya and carrots for treating inflammatory diseases. Table 4 shows the case studies of carrot and papaya extract outcomes.

**TABLE 4:** Preclinical and in vitro evidence on the biological effects of carrot and papaya extracts in immune modulation and health outcomes.

Category	Study	Key findings	Mechanistic pathways	Clinical implications	References
Carrot extracts	Phytochemical composition analysis	Rich in carotenoids, polyphenols, vitamins (antioxidant/anti‐inflammatory)	Scavenges ROS via Nrf2/ARE pathway	Supports dietary interventions for chronic inflammation	Yusuf et al. (2021), Sharma et al. (2012), Que et al. (2019), Roy et al. (2022)
	Agroecological impact on bioactives	Carotenoid/flavonoid levels vary with growing conditions	Modulates MAPK/NF‐κB signaling	Highlights need for standardized cultivation	Koudela et al. (2021), Schulzova et al. (2022)
	Antioxidant/anti‐inflammatory effects	Free radical scavenging; reduces TNF‐α, IL‐6 in vitro	Inhibits COX‐2 and iNOS expression	Potential adjunct in inflammatory diseases	Mizgier et al. (2016), Blando et al. (2021), Agarwal and Shanmugam (2022)
	Bioactivity of colored carrots	Black/purple varieties show higher antioxidant activity than orange	Anthocyanins modulate NLRP3 inflammasome	Functional food development for oxidative stress	Bhandari et al. (2022), Mandrich et al. (2023)
	Dietary roles in immunity	Enhances immune responses; reduces oxidative stress biomarkers	Boosts T‐cell activity; ↑ glutathione levels	Immune support in aging/viral infections	Childs et al. (2019), Calder et al. (2020), Patil et al. (2021)
Papaya extracts	Bioactive compound analysis	Contains papain, flavonoids, carotenoids (antioxidant/anti‐inflammatory)	Papain degrades proinflammatory cytokines	Wound healing; anti‐aging formulations	Khor et al. (2021), Al‐Seadi et al. (2021), Chandrasekar (2020)
	Anti‐inflammatory effects	Reduces TNF‐α, IL‐6 in vitro and in vivo	Suppresses NF‐κB and STAT3 pathways	Management of autoimmune conditions	Jeon et al. (2022), Soib et al. (2020), Pathak et al. (2022)
	Antidiabetic properties	Hypoglycemic effects via insulin sensitization	Activates AMPK/PPAR‐γ pathways	Adjuvant therapy for type 2 diabetes	Agada et al. (2021)
	Wound healing and cytoprotection	Promotes keratinocyte migration; reduces oxidative damage	↑ TGF‐β and collagen synthesis	Topical applications for burns/ulcers	Niveditha et al. (2020), Nguyen et al. (2016)
	Immunomodulatory effects	Enhances adaptive immunity via cytokine modulation	↑ IL‐10; ↓ IL‐1β	Vaccine adjuvants; post‐infection recovery	Olayem et al. (2024), Fatima et al. (2020), Alhazmi et al. (2021)
	Impact on viral infections	Improves platelet counts in viral fever	↑ Thrombopoietin receptor signaling	Supportive care in dengue/COVID‐19	Heena and Sunil (2019), Uddin et al. (2023)
	Carotenoid bioavailability	High bioavailability supports skin/eye health	↑ Retinol conversion in enterocytes	Nutraceuticals for macular degeneration	Laurora et al. (2021), Deis et al. (2021)

### 6.2 Limitations and Research Gaps

There is insufficient research on papaya and carrot extracts' anti‐inflammatory and immune‐modulating properties in many areas, even with encouraging preclinical and clinical data. Among the clinical studies, variability in study designs, dosages, preparation techniques, and characteristics of participants is a significant drawback. Therefore, formulating uniform standards for their application in clinical practice is challenging (Akbari et al. 2022). Moreover, although valuable information is obtained through research conducted on animals and in vitro, extrapolation of these findings to human populations continues to be challenging because many studies fail to provide long‐term data on the safety and efficacy of extracts of papaya and carrot (Miao et al. 2024; Hosseini et al. 2018). In terms of confirming the effectiveness of these extracts in various populations of patients and determining possible interactions with other medications or treatments, further well‐conducted, randomized clinical trials are needed (Roy et al. 2022).

## 7 Challenges and Future Directions

One of the greatest issues with using plant extracts such as papaya and carrots in therapeutic usage is standardization. Carotenoids within carrots and papayas, for illustration, may comprise tremendously fluctuating bioactive constituents because of fluctuating cultivar, ripening eras, cultivation performances, and processing (Chauhan et al. 2020). Because of such differences, reproducibility and predictability of beneficial effects are compromised. The inability to find effective prescription regimens because of the deficiency of set quantities of active elements can limit the therapeutic usage of these extracts. Clinical solicitation needs quality regulators and greater standards because dissimilar extraction and preparation approaches can produce different safety and efficacy profiles (Akbari et al. 2022).

To authenticate the anti‐inflammatory and immune‐controlling activities of carrot and papaya extracts in humanoid populations, greater, more vigorous scientific trials are desirable, even with auspicious outcomes in preclinical investigations. Generalizability is delayed in most clinical investigations due to causes like small illustration sizes, short examination durations, and deprived demonstration of contributors (Hosseini et al. 2018). Additionally, most of the investigation that has been done on these extracts has been focused on metabolic illnesses or rheumatoid arthritis, with little consideration paid to the far‐reaching inferences on immune function and systemic inflammation. To evaluate the therapeutic worth of these extracts, randomized, double‐blind, placebo‐controlled investigations with lengthier follow‐up intermissions and standardized yields across different populations need to be assumed (Cicio et al. 2022).

### 7.1 Mechanistic Studies, Bioavailability, and Novel Applications

Future investigations on the extracts of papaya and carrot should emphasize insufficient significant areas to recover their clinical effectiveness. First, mechanistic investigation requires being showed to understand the mechanisms of action among the bioactive constituents in these extracts and the molecular and cellular paths concerned in immune regulation and inflammation. For instance, the influence of carrot carotenoids and polyphenols on oxidative stress, cytokine fabrication, and immune cell signaling could be investigated Roy et al. (2022). Another significant factor limiting the medicinal efficacy of plant‐derived extracts is bioavailability. Many bioactive compounds in vitro have their effectiveness in vivo compromised by poor absorption in the gastrointestinal wang et al. (2024). Therapeutic outcomes from studies exploring strategies to enhance the bioavailability of papaya and carrot extracts, for instance, by coadministration with other bioenhancers or by preparation of nanoformulations, will be improved. Finally, new applications of these extracts must be explored. Papaya and carrot extracts possess immunomodulatory and anti‐inflammatory activity. Nonetheless, they do have future scope in other long‐term ailments accompanied by inflammation, including cancer, cardiovascular disease, and neurological conditions. Pharmaceutical drugs, functional foods, and nutraceuticals are likely future applications of these plant extracts, as is being studied now (Chandrasekar 2020; Uddin et al. 2023).

## 8 Conclusion and Future Trends

Papaya and carrot root extracts contain potential anti‐inflammatory and immunological response‐stimulating properties. Vitamins, carotenoids, and polyphenols are only a few of the bioactive composites present in plenty in the vegetable extracts that significantly contribute to modulating inflammation and immunological reactions. The enzymatic action of papaya extracts and the antioxidant activity of carrot root contribute to the therapeutic efficacy of the two. Steady dosage, safety, and long‐term consequences demand further trials with clinical investigations, even though the preclinical trials have revealed promising results. The synergistic effects of these extracts when jointly present new exciting avenues for the creation of nutraceuticals and functional foods aimed at treating inflammatory diseases and the immune system.

Although many challenges need to be overcome, using papaya and carrot root extracts in immune modulation and treatment of inflammation seems promising. First, since the preparation techniques may alter the potency and efficiency of the bioactive compounds, the extracts need standardization to ensure uniformity in their clinical application. Large, representative populations must undergo rigorous clinical trials to confirm the health benefits identified in preclinical studies. Future research should also focus on discovering how the bioactive compounds in papaya and carrot root extracts specifically exert their biological activity, particularly their interaction at a cellular and molecular level to affect inflammation and the immune response. More research on increasing the bioavailability of these substances, such as innovation in formulations or administration, may further increase their therapeutic potential. Investigating novel applications of these extracts in chronic diseases where inflammation plays a central role, such as cancer, heart diseases, and neurological diseases, could also increase their therapeutic potential. Additional research in these directions may birth novel plant‐based treatments for different inflammatory and immune‐related disorders.

## Author Contributions

**Muhammad Tayyab Arshad:** methodology (equal), writing – original draft (equal). **Sammra Maqsood:** data curation (equal), writing – review and editing (equal). **Ali Ikram:** supervision (equal), validation (equal). **Muhammed Adem Abdullahi:** formal analysis, Conceptualization

## Disclosure

Institutional Review Board Statement: The authors have nothing to report.

Informed Consent Statement: The authors have nothing to report.

## Conflicts of Interest

The authors declare no conflicts of interest.

## Data Availability

The data supporting this study's findings are available from the corresponding author upon reasonable request.
